# Motivationspsychologen als politische Aktivisten: Zur Politik der Selbststeuerung in den USA der langen 1960er Jahre

**DOI:** 10.1007/s00048-022-00348-5

**Published:** 2022-10-24

**Authors:** Lukas Held

**Affiliations:** Universität Zürich, Zürich, Schweiz

**Keywords:** Motivationspsychologie, Entwicklungspolitik, Neoliberalismus, Unternehmer-Wissenschaftler, Antipolitik, Motivation psychology, Development politics, Neoliberalism, Scientific political activism, Consulting, Anti-politics

## Abstract

Der Artikel analysiert am Beispiel des amerikanischen Motivationspsychologen David C. McClelland, wie Psychologen in den langen 1960er Jahren über die Verbreitung ihres Wissens als großzügige Wissensspender mit gesellschaftspolitischer Agenda auftraten, ohne dabei das Feld der institutionalisierten Politik betreten zu müssen. Untersucht wird eine vermeintlich passive Form von Aktivismus jenseits von Lobby- und Beratertätigkeit, die sich den „Umweg“ über die Politik zu ersparen hoffte, indem sie Bürger zur Selbststeuerung animierte, um damit Veränderungen herbeizuführen, die angeblich jenseits strukturverändernder Maßnahmen lagen. Anhand zweier Fallbeispiele wird argumentiert, dass sich das häufig als „neoliberal“ eingeordnete Prinzip der Selbststeuerung parallel zur strukturverändernden Planungspolitik entwickelte und nicht erst deren Scheitern voraussetzte. Weil dies häufig auf unternehmerischem Weg geschah, führte dies in der Psychologie zur Entstehung des Unternehmer-Wissenschaftlers, der Wissenschaft, Training und gesellschaftliche Reform als Geschäft betrieb. Anders als die politische Einflussnahme durch Lobbyismus war diese Form des scientific political activism zwar *direkt*, insofern unmittelbar auf einzelne gesellschaftliche Gruppen eingewirkt wurde, hinsichtlich ihrer Erfolgsaussichten aber auch *prekär*, weil die Frage, ob die Adressierten das ihnen Angebotene für sich zu übernehmen bereit waren, von Bedingungen abhingen, auf die die beteiligten Wissenschaftler keinen Einfluss hatten. Schon in den langen 1960er Jahren, so das Fazit, waren Programme, die auf die Aktivierung des Individuums setzten, Teil dessen, was wir als staatlich gesteuerte Wohlfahrtspolitik erinnern.

## Abstract (en)

Using the example of the American motivational psychologist David C. McClelland, this article analyses how psychologists in the long 1960s acted as generous purveyors of knowledge in order to bring about far reaching social change, without having to enter the field of institutionalised politics. The article thus explores a supposedly passive form of activism beyond lobbying and consultating that was intended to encourage citizens to self-direct in order to bring about changes that were supposedly beyond the reach of structural planning policy. Using two case studies, it is argued that the principle of self-direction often associated with neoliberalism developed in parallel with planning policy in the long 1960s, rather than as a consequence of its failure in the 1970s and 1980s. Since this was often done in an entrepreneurial way, it led to the emergence of the entrepreneurial scientist who ran science, educational training and social reform as a business. Unlike political influence through lobbying, this form of scientific political activism was *direct* in that it directly impacted individual social groups, but it was also *precarious* in its chances of success, because whether those approached were willing to accept what was offered to them depended on conditions over which the scientists involved had no control. As early as the long 1960s, it is concluded, privately run programmes aimed at activating the individual were part of what we know as state-directed welfare policy.

Im September 1969 verkündete der amerikanische Kognitionspsychologe George A. Miller in seiner Presidential Address vor der American Psychological Association (APA): „Our responsibility is less to assume the role of experts and try to apply psychology ourselves than to give it away to the people who really need it—and that includes everyone“ (Miller 1969: 1071). Unter dem vollmundigen Titel „Psychology as Means of Promoting Human Welfare“ explizierte Miller eine Strategie der gesellschaftlichen Einflussnahme, die seinen Kolleg*innen weder die Rolle des Experten, noch die des Lobbyisten zuwies, sondern die des großzügigen Wissensspenders, der Werkzeuge zur Selbsthilfe zu vergeben hat. Paradox formuliert zielte sein Vorschlag zur gesellschaftlichen Einflussnahme auf eine Politik, die sich den „Umweg“ über die Politik erspart. Statt Politiker*innen dazu zu bringen, bestimmte Entscheidungen zu treffen, die dann in gesetzliche Regelungen münden, sollte der Bürger direkt erreicht und zur Selbststeuerung ermächtigt werden. Gesellschaftliche Veränderungen via Politikberatung voranzubringen, sei für die Psychologie nicht so sehr deshalb der falsche Weg, weil sich die APA ihrer eigenen Satzung nach nicht als politische Interessengruppe verstand, sondern weil auf dem institutionellen Weg kaum etwas erreicht werden könne. „There is no possibility of legislating the changes I have in mind“, gab Miller freimütig zu verstehen. „Passing laws that people must change their conceptions of themselves and others is precisely the opposite of what we need“ (ebd.: 1072). Mit anderen Worten: Nicht um Strukturveränderung sollte es gehen, sondern um die Vermittlung von Strategien zur Selbstmobilisierung Einzelner, die anzuwenden dann in deren eigenem Ermessen läge, statt staatlich verordnet zu sein. Wer behaupte, Psychologen stünde zur Beeinflussung der Gesellschaft nur die Rolle des beratenden Experten offen und soziale Reformen könnten nur das Ergebnis von *politischen* Entscheidungen sein, schränke ihren Wirkungsbereich unnötig ein. „This presupposition should not go unchallenged“, ließ Miller seine Zuhörer*innen wissen. „Perhaps our options for promoting human welfare are broader than this debate would suggest“ (ebd.: 1065). Worauf es jetzt ankomme, sei, „more active participation by individual psychologists“ (ebd.).

Miller gab damit 1969 als programmatische Losung aus, was der amerikanische Motivationspsychologe David C. McClelland bereits Ende der 1950er Jahre im Rahmen entwicklungspolitischer Debatten vorgeschlagen hatte und seit den frühen 1960er Jahren in Eigenregie auch praktizierte. McClelland forschte seit 1956 an der Harvard Universität und gründete 1963 seine eigene Beratungsfirma. Im Folgenden stehen seine Bestrebungen im Zentrum, durch die spezifisch unternehmensberaterische Aufbereitung und Verbreitung seines Wissens umfassende gesellschaftliche Veränderungen anzustoßen. Diese Aufbereitung und aktive Verbreitung wissenschaftlichen Wissens bezeichne ich als Aktivismus, weil es sich dabei um den Versuch handelte, nicht etwa nur ausgewählte Gruppen oder lokal begrenzte Bereiche zu beeinflussen, sondern die Gesellschaft als solche, und zwar gleichsam in Eigenregie hinter dem Rücken der Politik. Im darin zu beobachtenden generösen Selbstverständnis (Wissensweggabe als Großzügigkeit), im Fehlen sowohl einer konsensgestützten Autorisierung wie auch einer sozialen Bewegung als Trägerschaft lassen sich Ähnlichkeiten zu bestimmten Formen philanthropischen Engagements erkennen, das in der Forschung dann als politische Betätigung verstanden wird, wenn es sich auf die Förderung ganzer Politikfelder (etwa die Bildung) richtet, statt nur auf einzelne Institutionen (etwa eine einzelne Schule) (Bernholz et al. 2016; Reich 2018: 15 ff.). Im Unterschied zur Settlement-Bewegung des späten 19. und frühen 20. Jahrhunderts ging es bei diesem Engagement nicht um die Herstellung von Gemeinschaft durch den Abbau von Klassenschranken (vgl. Landhäußer 2009: 31–85), sondern um die Forcierung wirtschaftlichen Wachstums. Und im Unterschied zu den Aktivierungsprogrammen des Taylorismus war das Ziel nicht Effizienzsteigerung durch Rationalisierung von Produktionsabläufen, sondern die Einübung von Techniken zur Selbstmobilisierung jenseits industrieller Strukturen (vgl. Baker 2021).

Versuche, gesellschaftliche Probleme durch psychologische Modellierungen Einzelner zu lösen, sind aus politiktheoretischer Perspektive als eine Art Antipolitik analysiert worden, die die „eigentliche“ Politik der gesellschaftlichen Willensbildung unterlaufe und bedrohe.^1^ Jedoch sollte nicht übersehen werden, dass das Eintreten für psychotechnische Mittel zur Lösung gesellschaftlicher Probleme auch selbst politisch sein konnte. In seinen Bedingungen und Wirkungen ist es damit auch prekär sowie historisch kontingent. Denn es sind die zeit- und ortsspezifischen Rahmenbedingungen (im Folgenden vor allem die entwicklungspolitische Lage im Kalten Krieg und der gouvernementale Krisendiskurs der 1960er Jahre), die darüber entscheiden, ob ein derartiger Aktivismus überhaupt unternommen wurde, ob er auf Resonanz stieß und ob er sich verstetigte. Das Funktionieren der vorgeschlagenen Programme oder die wissenschaftliche Güte der lancierten Konzepte allein reichten dafür nicht.

Bevor ich genauer auf McClelland selbst eingehe, ist darum ein Blick auf die Deutung nötig, die zu den „langen 1960er Jahren“ in Bezug auf das Verhältnis von Planung und Selbststeuerung entwickelt worden sind, um darin das Aufkommen von McClellands spezifischem Aktivismus angemessen einordnen zu können. In politikgeschichtlicher Hinsicht beschreibt die ältere Forschung die 1960er Jahre als Jahrzehnt der gesellschaftlichen und ökonomischen Strukturveränderungen, nicht aber als Jahrzehnt der Selbststeuerung (mit Blick auf die Gesellschaft werden die 1960er Jahre natürlich als Jahrzehnt der Proteste beschrieben, vgl. Rossinow 2018). Das trifft sowohl für Westeuropa zu, wie auch für die USA, wo die Zeit zwischen dem Regierungsantritt von John F. Kennedy 1961 und dem Ende der Präsidentschaft Lyndon Johnsons 1969 nach dem New Deal eine zweite Phase großer innen- und außenpolitischer Pläne und Reformen war (Doering-Manteuffel & Raphael 2012: 39–45; van Laak 2008; Doering-Manteuffel 2008; Metzler 2005; Ruck 2000; Agar 2008; Marwick 1998). Man denke an Kennedys 1961 verkündete *decade of development* und Johnsons drei Jahre später versprochene *Great Society*. Dirk van Laak nannte die 1960er Jahre gar die „historische Sattelzeit der Planung“ (van Laak 2008: 325). Planung habe dieser Deutung zufolge immer aufs Ganze der Gesellschaft gezielt, auf „Gesellschaftspolitik“, was sowohl einen alles überwölbenden starken Staat, als auch einen impliziten Antiindividualismus vorausgesetzt habe, insofern es eben um die Veränderungen der grundlegenden Strukturen und nicht um die Veränderung einzelner Individuen ging (Metzler 2005: 794; Ruck 2000: 385 f.; Doering-Manteuffel 2008: 404; Doering-Manteuffel & Raphael 2012: 42).^2^ Die Idee einer aufs Individuum zielenden Politik der Selbststeuerung wird darum gemeinhin nicht mit den 1960er Jahren, sondern eher mit den späten 1970er, ja den 1980er Jahren in Verbindung gebracht, genauer noch, mit dem Aufstieg dessen, was man Neoliberalismus nennt. Ihr „Durchbruch“ sei gerade eine Antwort auf das Scheitern der großen Planungsutopien gewesen und im Zusammenhang mit einer neuen „Krise des Regierens“ (Leendertz 2019; Leendertz 2016) und einem sich daraufhin nun wieder zurückziehenden, zur Eigeninitiative aufrufenden Staat zu sehen.^3^

So plausibel diesem Narrativ zufolge die These scheint, die Politik der Selbststeuerung sei auf die Politik der Strukturveränderung *gefolgt*, so sehr muss doch die Annahme eines linearen Nacheinanders vor dem Hintergrund neuerer Forschung relativiert werden. Und zwar sowohl in Bezug auf das „Jahrzehnt der Modernisierungstheorie“ (Kunkel 2008: 155), die 1960er Jahre, wie auch in Bezug auf den sogenannten „Durchbruch“ des Neoliberalismus. „Narratives centred around breaks“, betonen die Autoren eines neuen Forum-Beitrags im *Journal of Modern European History *zum Konzept des Neoliberalismus in der Zeitgeschichte, „inevitably tend to homogenize the eras before and after an alleged rupture“ (Bänziger et al. 2019: 382). Konzepte wie „Trente Glorieuses“ (Jean Fourastié), „Golden Age“ (Eric Hobsbawm) oder „Nachkriegsboom“ (Doering-Manteuffel & Raphael), mit denen die ersten drei Nachkriegsjahrzehnte häufig gefasst werden, lassen den Eindruck einer Harmonie, einer Zeit frei von Krisen und Widersprüchen entstehen, die in Wirklichkeit kaum existierte (Tooze 2019).

Daniel Immerwahr hat in seiner Studie *Thinking Small* (Immerwahr 2015) für die USA argumentiert, dass *parallel* zum Aufkommen der gesellschaftsumspannenden Modernisierungstheorien ein Entwicklungsdenken Verbreitung fand, das auf der Ebene der Gemeinschaft ansetzte und ausgewählte Einzelne zu adressieren versuchte. Die Betonung von Freiwilligkeit, Eigeninitiative und Selbstverantwortung in diesen Ansätzen sowie die Tatsache, dass neben staatlichen Institutionen häufig private Unternehmen als ausführende Mittler „community development“ betrieben, hat Amy C. Offner zum Anlass genommen, den Übergang von der Hochphase des amerikanischen Wohlfahrtsstaats der 1950er und 1960er Jahre zum sogenannten Neoliberalismus der 1970er und 1980er Jahre gerade nicht als Durchbruch von etwas ganz Neuem, nur ideologisch von langer Hand Vorbereitetem zu interpretieren, sondern vielmehr als fortsetzende Vereinseitigung jener unternehmerischen Elemente, die schon die *mixed economy* der Eisenhower‑, Kennedy- und vor allem der Johnson-Jahre ausgezeichnet habe, ohne dass die involvierten Akteure sich selbst deshalb als „Neoliberale“ verstanden oder auf das ideologische Angebot der Mont Pèlerin Society zurückgegriffen hätten. „By design and as a point of pride“, schreibt Offner, „every project of purported ‚state-led‘ development was in equal measure a private initiative“ (Offner 2019: 17). Neuere Forschungen zu den Regierungstechniken staatssozialistischer Gesellschaften weisen zudem darauf hin, dass bislang in der Literatur als „neoliberal“ eingeordnete Regierungstechniken wie Responsibilisierung und Selbststeuerung auch Teil einer „sozialistischen Gouvernementalität“ waren, nicht zuletzt, weil sich hier Planungsdenken und Kybernetik bereits in den 1960er Jahren verschwisterten (Palmer & Winiger 2019; Arend 2019).

Planungspolitik, die auf Strukturveränderung zielte, und die Politik der Selbststeuerung, die auf die Initiative Einzelner setzte, so möchte ich daran anschließend im Folgenden argumentieren, sollten darum nicht als Nacheinander gedacht, sondern müssen als gleichzeitiges Nebeneinander analysiert werden. Sie lösten nicht einander ab, sie griffen ineinander. ‚Selbststeuerung‘ war nicht nur ein linkes Prinzip der Abkehr von allen Strukturen und Autoritäten, von institutionalisierter Politik überhaupt, wie zum Beispiel für die Kinderladenbewegung, für die *counterculture* und mit Verweis auf die Humanistische Psychologie überzeugend argumentiert worden ist (Grogan 2013: 143–208; Reichardt 2014: 721–781; Tändler 2016: 249–281, besonders 270–279). Sie war selbst eine Regierungstechnik zur Sicherung des Status quo, die schon in den „langen 1960er Jahren“ eine nicht-linke Politikgeschichte hat. Wissenschaftler, genauer gesagt Motivationspsychologen (es handelte sich überwiegend um Männer), spielten darin eine wichtige Rolle. Konkret auf die hier im Zentrum stehende Fallstudie bezogen lautet die Frage: Wie versuchte McClelland mit seiner Forschung Gesellschaft zu gestalten, warum tat er dies hinter dem Rücken der Politik gleichsam auf eigene Faust als Unternehmer-Wissenschaftler und welche Schlüsse lassen sich daraus für die Geschichte des Neoliberalismus ziehen?

In einem ersten Schritt werde ich zeigen, wie McClelland sein theoretisches Wissen praktisch anwendbar machte und anschließend anhand seines ersten entwicklungspolitischen Einsatzes in Indien analysieren, warum er die Durchführung seiner Programme schon bald als Unternehmer betrieb statt als beratender Experte. Im dritten Abschnitt fokussiere ich auf die gesellschaftspolitischen Rahmenbedingungen innerhalb der USA, unter denen politisch umstrittene Fragen als psychologisch lösbare Probleme erscheinen konnten. Abschließend versuche ich das Beispiel ‚McClelland‘ im Hinblick auf das in der Forschung vorherrschende Verständnis der langen 1960er Jahre zu deuten.

## Testinstrumente als Trainingswerkzeuge

Der in Mount Vernon, New York, geborene David C. McClelland (1917–1998) verstand sich noch Mitte der 1950er Jahre als rein theoretisch arbeitender Psychologe, wie er 1971 rückblickend in einem Interview mit* Psychology Today* bemerkte (Harris 1971: 71). Sein Forschungsfokus an der Wesleyan Universität im Bundesstaat Connecticut, an die er nach seiner Promotion in Yale als Assistenzprofessor zuerst berufen wurde, hatte nach dem Zweiten Weltkrieg auf der Entwicklung eines Testinstruments gelegen, mit dem Bedürfnisse objektiv messbar gemacht werden sollten. Die Ausgangsfrage, ob Bedürfnisse wie etwa Hunger oder Sex die Wahrnehmung verzerren können, war im Zusammenhang des zurückliegenden Krieges aufgekommen und die zu diesem Zweck vom Office of Naval Research ab 1947 finanzierte Grundlagenforschung ursprünglich von militärischem Interesse gewesen. Das bis Anfang der 1950er Jahre von McClelland und seinem Forscherteam entwickelte Testverfahren ermöglichte nicht nur physiogenetische Bedürfnisse, sondern auch psychogenetische Motive wie Angst und Neugier in ihrer Stärke zu bestimmen. Damit rückte schon bald ein psychologisches Motiv ins Zentrum seines Interesses, das sich in forschungspragmatischer Hinsicht besonders leicht evozieren ließ und zugleich kulturell von herausgehobener Bedeutung zu sein schien. Gemeint war das Streben, eine Sache besonders gut zu machen und das eigene Tun an einem Gütemaßstab auszurichten, um es als gelungen oder gescheitert zu bewerten. McClelland nannte es das „Leistungsbedürfnis“ („need for achievement“, kurz „n Ach“) und veröffentlichte die erste Ergebnisbilanz seiner Forschung 1953 als Sammelband unter dem Titel „The Achievement Motive“ (McClelland et al. 1953).

Als die Harvard Universität ihm im Sommer 1956 einen von der Ford Foundation finanzierten Lehrstuhl für Persönlichkeitspsychologie anbot, tat sie das weniger auf der Basis dieser ersten großen Veröffentlichung, als vielmehr deshalb, weil McClelland in Aussicht stellte, seine motivationspsychologische Grundlagenforschung entwicklungspolitisch fruchtbar zu machen. Einerseits hatten seine kulturvergleichenden Messungen nämlich gezeigt, dass das „Leistungsbedürfnis“ universell vorhanden sei – anscheinend ein menschliches Grundvermögen wie Laufen und Sprechen. Andererseits hatte dessen mal stärkere, mal schwächere Ausprägung in unterschiedlichen Ländern gezeigt, dass es sich um eine Variable handelte, deren Entwicklung von der elterlichen Erziehung abzuhängen schien. Je früher Eltern ihre Kinder zur Selbständigkeit erzogen (zugleich aber auch nicht *zu* früh), desto leistungsorientierter erwiesen sich ihre Heranwachsenden. Weil McClelland diesen Befund mit Max Webers berühmter These parallelisierte, zwischen protestantischer Lebensführung und kapitalistischer Wirtschaftsentwicklung bestehe ein enger Zusammenhang, leitete er analog die wirtschaftliche Entwicklung eines beliebigen Landes aus der durchschnittlichen Leistungsmotivation seiner Bevölkerung ab. „[I]f we can measure it [= need for achievement in a culture],“ schrieb er im August 1955 in einem Förderantrag für die Ford Foundation, „we are in a position to predict, for example, whether a given country is a ‚good risk‘ as far as economic aid is concerned in terms of its value or need structure. … [T]his … should enable us to pick nations … that are most likely to respond successfully to economic aid.“^4^ Mit anderen Worten: McClelland argumentierte, Entwicklungshilfe lohne sich besonders dann, wenn in der Bevölkerung des zu fördernden Landes die nötige Leistungsmotivation vorhanden wäre, weil die gebotene Hilfe nur dann tatsächlich aufgegriffen und sich als unternehmerische Aktivität verstetigen und so schließlich zu wirtschaftlichem Wachstum führen werde. In einer Zeit, als die Eisenhower-Administration versuchte, im sich verschärfenden Wettbewerb des Kalten Krieges um die damals so genannte „Dritte Welt“ buchstäblich Land zu gewinnen, trafen derartige Ankündigungen und Versprechen auf offene Ohren (vgl. Lorenzini 2019: 52–67; Ekbladh 2010: 182–189). Überzeugt von McClellands Antrag hielt das Gutachtergremium der Ford Foundation in einem internen Bewilligungsentscheid fest: „Findings of the study should be directly applicable in the economic development programs that have become important in the modern world.“^5^

An diesem Punkt hatte sich McClelland’s Interesse von psychologischer Grundlagenforschung auf entwicklungspolitische Anwendungsfragen verschoben. Der eigentliche, entwicklungspolitisch relevante Durchbruch erfolgte jedoch erst, als er auf die Idee kam, das von ihm entwickelte Messverfahren zugleich zur Stärkung des „Leistungsbedürfnisses“ einzusetzen. Ende der 1960er Jahre bemerkte er dazu in einem Artikel lapidar: „[T]here is no reason why any testing device cannot be turned into a teaching device“ (McClelland 1969b: 10 f.). Wer wisse, wie ein spezifischer psychologischer Test funktioniere – gleich ob Intelligenz- oder Leistungsmotivationstest –, könne die zu Testenden darin unterweisen und so dazu bringen, die betreffende Eigenschaft zu trainieren. Während die Mehrheit der akademischen Psychologen in den USA in den 1950er Jahren die Erlernbarkeit von Motiven (in McClellands Sinn) bei Erwachsenen für nahezu unmöglich hielt und einige die Wirksamkeit von Psychotherapien empirisch widerlegt zu haben meinten,^6^ begann McClelland mit seinem Plädoyer für die Steuerbarkeit der Persönlichkeit hervorzutreten. In gewisser Weise hatte er diesen Weg bereits mit der behavioristischen Grundannahme eingeschlagen, alle Bedürfnisse seien erlernt, denn alles Erlernte ist grundsätzlich veränderbar. Damit war die Möglichkeit zur Steuerbarkeit bereits in McClellands Motivationstheorie angelegt. Dass es ausgerechnet ein Schüler des Behavioristen B. F. Skinner war, der 1958 in seiner Dissertation als erster versuchte, die Leistungsmotivation von Schüler*innen zu steigern, indem er sie durch künstlich hergestellte Wettbewerbssituationen schlicht hervorrief und dann belohnte, verwundert nicht.^7^ Das Prinzip der klassischen Konditionierung hatte Ende der 1950er Jahre als theoretische Grundlage zur Entwicklung sogenannter *teaching machines* gedient, von denen sich insbesondere nach dem Sputnik Schock Bildungsreformer in den USA (und darüber hinaus) die Revolutionierung des Schulunterrichts erhofften und Ökonomen technologische Innovationen und wirtschaftliches Wachstum.^8^ „Because of the learning-theory argument by Skinner’s student,“ erklärte McClelland seine Hinwendung zu angewandten Problemstellungen im eingangs erwähnten *Psychology Today* Interview, „I was getting interested in the idea of training courses to develop achievement motivation“ (Harris 1971: 74).^9^

Die erste Möglichkeit, dies auszuprobieren, bot sich ihm bezeichnenderweise im Sommer 1961 in Mexiko City, wo er ein Jahr zuvor während eines Sabbaticals dem Unternehmensberater Elliott R. Danzig begegnet war, der ihm anbot, sein motivationspsychologisches Wissen zu adaptieren. „I do this kind of training all the time“, habe dieser ihm gesagt, „Why don’t I just adapt what I do and put this stuff in?“ (Harris 1971: 74). Unterstützt durch einen kleinen Förderbetrag der Carnegie Corporation erhielt McClelland im August 1961 so seine ersten Probeläufe, Unternehmer zu trainieren (ebd.). Weitere Pilotstudien erfolgten im Folgejahr mit der Textile Industry’s Research Association in Ahmedabad in Indien, mit der Nippon Management Association in Japan sowie mit der IBM Corporation in den USA.^10^ Die Programme bestanden aus einer Theoriephase, in der die Teilnehmenden mit McClellands Verfahren zur Messung des Leistungsmotivs bekannt gemacht wurden. In der darauffolgenden Praxisphase galt es dann, kurze Geschichten zu schreiben, die so viele Leistungsmotive enthalten sollten wie möglich, das heißt Formulierungen und Beschreibungen, in denen ein Sich-Anstrengen, aus eigenem Antrieb Handeln, Gut-und-effizient-sein-Wollen zum Ausdruck kam. Über mehrere Tage gestreckt und mit wiederholten Selbstreflexions- und Planungssessions gekoppelt sollten die Teilnehmenden so mit der Zeit „Achievement Thinking“ erlernen, demgemäß sie schließlich handeln würden.^11^

Die Ergebnisse seiner Pilotstudie bestärkten McClelland in der Überzeugung, nun tatsächlich in der Lage zu sein, Menschen zur effizienteren Selbststeuerung anleiten zu können. Von nun an sah er sich gerüstet, sein erstes eigenes Entwicklungsprojekt auf den Weg zu bringen. „It is our belief based on these pilot training experiences“, schrieb er im Januar 1963 in einem Forschungsantrag für die nur zwei Jahre zuvor von John F. Kennedy gegründete Agency for International Development (AID), „that our type of intensive psychological treatment of motives, values, and self-development really ‚gets through‘ to the managers and changes them […].“^12^

Bezeichnend war dieses Vorgehen deshalb, weil in den 1950er Jahren nicht wenige amerikanische Sozialwissenschaftler*innen in den Globalen Süden ausschwärmten, um als Entwicklungshelfer die Tauglichkeit ihrer Theorien zu erproben. Viele kehrten Mitte der 1960er Jahre in die USA zurück, nur um dann in Johnsons groß angelegtem *War on Poverty* ihre gesammelten Erfahrungen und geknüpften Kontakte als Dienstleistungen anzubieten (Offner 2019: 1–18, 175–274). Die Gleichsetzung armer Länder mit armen Regionen in den USA hatte ein Betätigungsfeld geschaffen, das für eine Vielzahl amerikanischer Entwicklungsakteure nun die Möglichkeit bot, in der eigenen Heimat aktiv zu werden (ebd.: 180). Die Frage ist, warum viele – so auch McClelland – das als Unternehmer-Wissenschaftler taten, und nicht als beratende Experten, als vom Profitinteresse ungetrübte Wissenschaftler*innen. Die naheliegende Antwort, weil sich mit der Gründung einer eigenen Beratungsfirma sehr viel Geld verdienen ließ, greift hier zu kurz.

## Unternehmerische Wissenschaft

Eine Erklärung für McClellands Wandel zum Unternehmer-Wissenschaftler muss bei der Erfahrung ansetzen, die er bis 1965 als Empfänger staatlicher und privater Fördermittel im Ausland hatte machen müssen. Steven Shapin hat das Konzept des „entrepreneurial scientist“ vor allem auf Wissenschaftler*innen in den Life Sciences und den technischen Wissenschaften bezogen und insbesondere mit dem Bayh-Dole Act von 1980 in Verbindung gebracht, der das Recht zur kommerziellen Verwertung staatlich geförderter Forschungsergebnisse landesweit einheitlich an die Universitäten übertrug. McClellands Hinwendung zur Privatwirtschaft erfolgte jedoch gut 17 Jahre vor dieser folgenreichen Grundsatzentscheidung (Shapin 2008: 209–217; Mowery et al. 2004). Schon Mitte der 1960er Jahre sollte er sich der Businesswelt als Unternehmer vorstellen, der im Rahmen seiner Beratertätigkeit auch Forschung betrieb. Und als jemand, der diese Forschung auf eine unpolitische Weise zu politischen Zwecken einzusetzen anbot – durch die Schulung Einzelner als Maßnahme zur Veränderung ganzer Gesellschaften. Im Vergleich zum politischen Aktivismus sozialer Bewegungen war das nicht weniger ambitioniert, im Unterschied zu diesen aber vermeintlich wertfrei, weil es nicht um die Vermittlung von Wertüberzeugungen ging, sondern um die Einübung von Denkroutinen.

Die Agency of International Development hatte McClelland im Dezember 1962 dazu ermuntert, sich um Mittel zu bewerben. McClelland hatte daraufhin im Januar des folgenden Jahres den oben erwähnten Antrag eingereicht und im Juni an der Harvard Business School eine Planungskonferenz abgehalten. In Absprache mit Repräsentanten „from various underdeveloped countries“ (McClelland & Winter 1969: 96 f.) entschied er dort, das in seinen Pilotstudien entwickelte Trainingsprogramm in Indien, Süditalien und Tunesien auf seine entwicklungspraktische Wirksamkeit zu testen. In Indien sollten Mitarbeiter mehrerer Textilfabriken geschult werden; in Süditalien gedachte man Unternehmensführer aus vier neapolitanischen Regionen zu trainieren; in Tunesien wollte man versuchen, Regierungsbeamte zu erreichen. Das Ziel: Über einen Zeitraum von mindestens fünf Jahren die tatsächlichen wirtschaftlichen Effekte des Trainings auf unternehmerischer Ebene (gemessen am Umsatz), auf regionaler Ebene (gemessen an der unternehmerischen Aktivität, zum Beispiel Unternehmensgründungen und -expansionen) und auf Landesebene (gemessen am nationalen Stromverbrauch) zu untersuchen.^13^ „We are ambitious enough“, schrieb McClelland in seinem AID-Antrag, „to want to try to turn a few slowly developing countries into achieving societies.“^14^ Anders als bei den zuvor durchgeführten Pilotstudien sollte es nun um ein mehrjähriges Engagement mit mehreren Trainings- und Evaluationsphasen gehen. Der Einsatz war weniger ein Forschungsprojekt zur Gewinnung neuen Wissens, als eine entwicklungspolitische Maßnahme, im Auftrag der neu geschaffenen Agency for International Development. Diese über seinen Lehrstuhl an der Harvard Universität abzuwickeln, erschien McClelland unpraktisch. Studenten wären für die anfallenden Aufgaben zu jung und zu unerfahren, Personal außerhalb von Harvard hätte nur über wissenschaftliche Mitarbeiterstellen beschäftigt werden können und diese wären somit zeitlich auf drei Jahre befristet gewesen (McClelland 1984: 24).^15^ Zunächst aus diesem rein organisatorischen Grund rief McClelland im Januar 1963 deshalb die *Human Resources Development Corporation* (HRDC) ins Leben, eine Beratungsfirma mit Sitz in Cambridge, Massachusetts (siehe auch Winter 1982: xii; McClelland 1984: 24).

Als er im Herbst zu einer mehrmonatigen Vortrags- und Vorbereitungsreise rund um die Welt aufbrach, erreichte ihn am 15. Oktober in Neu Delhi ein Brief von David E. Bell, dem Leiter der Agency for International Development, in dem dieser schrieb: „I have reluctantly concluded that we should not proceed with the financing of this project.“^16^ Ein Paukenschlag für McClelland, der in Indien bereits Mitarbeiter anzustellen begonnen und persönliche Auslagen über mehrere tausend Dollar getätigt hatte.

Mit einem Schlag vollständig ohne Finanzierung, war McClelland gezwungen, die für Süditalien und Tunesien geplanten Projekte zu streichen und für das verbleibende Projekt in Indien in aller Eile neue Gelder einzuwerben. Ein Antrag bei der Ford Foundation wurde ebenfalls ohne Begründung abgelehnt, ein weiterer bei der Carnegie Corporation in letzter Minute bewilligt. „The gap between what we had hoped to do and what we were actually able to do is obviously very great“ (McClelland & Winter 1969: 107), bekannte McClelland in seinem 1969 unter dem Titel *Motivating Economic Achievement* erschienenen Buch. In der Sache aber, so das dort präsentierte Ergebnis, hätten die schließlich in zwei Kleinstädten nahe Hyderabads durchgeführten Programme den erhofften Erfolg gehabt: „The aggregate effects of the courses include, to date, the mobilization of approximately Rs. 376,000 of specific new capital investments and about 135 new jobs. Measured by these figures, the courses certainly seem to have had an economic effect“ (ebd.: 231). Fortgeführt wurden sie nach Beendigung von McClellands Engagement in Indien aber nicht, weil der neue Direktor des Small Industries Extension Training Institut (SIET) in Hydarabad, das die Schirmherrschaft getragen hatte, nicht mehr länger daran glaubte (ebd.: 135). Als entwicklungspolitische Intervention, bilanzierte McClelland darum abschließend, „the project must be judged less than successful“ (ebd.: 364).

Interessant ist, welchen Schluss McClelland aus dieser Erfahrung zog. Schon der Umstand nämlich, dass er seinem Bericht ein eigenes Kapitel beifügte, in dem er die durchlebten organisatorischen Hürden ausführlich beschrieb, ist bemerkenswert. Offensichtlich bestand dessen Zweck darin, andere Wissenschaftler*innen vor einem ähnlichen Vorgehen geradezu zu warnen. „Because the research is carried out with the collaboration and support of government and semiprivate institutions“, ließ er gleich im Vorwort seine Leser*innen wissen, „the behavioral scientist often comes to feel that what happens is beyond his understanding and control“ (ebd.: viii). Auch wenn Gefühle wie Ärger und Frustration in einem wissenschaftlichen Bericht nichts zu suchen hätten, sah sich McClelland doch dazu gedrängt, diese in einige grundsätzliche Überlegungen zum Verhältnis von Wissenschaft und Politik zu kleiden. „Such emotions“, schrieb er, „do reflect some of the difficulties and complexities of coordinating scientific research with public institutions and policy-makers: such institutions have needs, standards, anxieties, and a logic of their own which often conflict with the requirements of getting research done“ (ebd.). Eine in guter Absicht getroffene Zusage sei „always subject to the caprice of political exigencies“, ohne dass sich eine Regierungsbehörde je für ihre Entscheidungen würde rechtfertigen müssen (ebd.). „Perhaps it can never be otherwise“, schloss McClelland frustiert, „for any institution that is involved with policy and society has to play by the rules of the political process if it hopes to survive“ (ebd.). In einem Briefentwurf an David E. Bell, den Direktor von AID, schrieb McClelland noch deutlich unumwundener: „I shall certainly advise my friends that they cannot believe what AID tells them, because however well intentioned it may be, AID is not free to live up to its own advice.“^17^ Diese Kritik traf nicht nur die Agency for International Development, sie traf auch die Ford Foundation.^18^

Dennoch gedachte McClelland nicht, seine angewandte Arbeit aufzugeben. Die Indien-Odyssee hatte ihm nämlich auch gezeigt, dass es einen Markt für seine Programme gab. Selbstbewusst schrieb er 1965 in der Dezember-Ausgabe des *Harvard Business Review* – eins der renommiertesten und meistgelesenen Management-Magazine in den USA –, die Nachfrage für seine Programme sei nun so groß, dass Bedarf für eine Organisation bestehe, „which can supply on a regular basis the kind of motivation training with which we have been experimenting in India“ (McClelland 1965b: 178). Zwar hatte McClelland, wie erwähnt, seine *Human Resources Development Corporation* in Wirklichkeit schon zwei Jahre zuvor ins Leben gerufen, nun aber schien ihm der Zeitpunkt gekommen, unter neuem Namen – *McBer & Company* – für deren Dienstleistungen zu werben. Mit klarer Anspielung auf Johnsons *War on Poverty* schrieb er weiter: „[T]here are plenty of opportunities around the poor that they are not exploiting. It is necessary to move in and increase their needs for achievement“ (ebd.).

Bemerkenswert ist, wie McClelland seine Firma auf diese Weise einerseits als Anbieter von Programmen pries, mit denen sich das Armutsproblem einer Lösung näherbringen lassen würde, andererseits aber auch die Notwendigkeit für weitere Forschung hervorhob, so als werde diese künftig von McBer & Company durchgeführt, nun aber „on a regular basis“ und damit mit mehr Stetigkeit als eine von staatlichen Fördergeldern abhängige Forschung. „Research on the achievement motive must and will continue“, hieß es gegen Ende seines Artikels. „We must discover far more precisely how to influence motivation and use that knowledge for human betterment (…)“ (ebd.). Die durchzuführenden Trainingseinheiten wären sowohl zu bezahlende Dienstleistungen als auch Experimente, deren Daten neues Wissen generieren. Die möglichen Anwendungs- und Einsatzfelder, die McClelland in seiner derart gefassten Doppelrolle als Unternehmer-Wissenschaftler sah, reichten von der Förderung leistungsschwacher Schüler*innen über strauchelnde Kleinunternehmer*innen bis hin zur Hilfe für Arbeitslose und Geisteskranke („the unemployed worker, the mentally sick“) (ebd.). Ohne näher zu erläutern, was genau er eigentlich meinte, als er abschließend schrieb, „even the political unwise are fair subjects to work on“, sah er sich in einem Anflug psychotechnischer Hybris sogar in der Lage, per Motivationstraining Bürgerkriege zu befrieden, so als beruhten diese in Ländern wie Vietnam oder dem Kongo auf einer Handvoll demotivierter Unruhestifter: „[T]here is no theoretical reason“, schrieb er, „why, once we understand the motives of participants in unstable governments such as South Vietnam or the Congo, we could not employ the same educational techniques to create a climate of political stability and enthusiasm for a country’s future“ (ebd.). Mit anderen Worten: Aufgaben, die zum Kernbereich von Politik gehören, wurden McClelland zum Geschäftsmodell. Leitend war dabei weniger das Interesse, Profit zu machen als seine Überzeugung, Gesellschaft auf beraterischem Weg effizienter zum Besseren verändern zu können als staatliche Politik. In diesem weitreichenden Veränderungsanspruch war McClelland als Unternehmer Aktivist, der zugleich auch Forschung betrieb – eine aus seiner Sicht weniger opportunistische denn pragmatische Rollenkombination.

*McBer & Company *sollte bis Anfang der 1960er Jahre Unternehmertraining unter anderem in Uganda, Tunesien, Ghana, Ecuador, Neu Guinea, Indonesien und auf der karibischen Insel Curaçao durchführen.^19^ Berichte über und Verweise auf dieses Engagement erschienen an prominenten Orten wie *TIME Magazine, Organizational Dynamics, Think* und *Forbes* (McClelland 1969a; Anonym 1969; McClelland 1969c; McClelland & Dowling 1972). „[E]ven Indian businessmen, a notoriously lethargic group, can be instilled with the spirit of entrepreneurship“, hieß es mit rassistischem Unterton in letzterem beispielsweise enthusiastisch. „[W]hat Dr. McClelland has to say could be helpful in choosing investments, in raising production and cutting costs, and in turning social dropouts into productive citizens“ (McClelland 1969a: 53). In diesem Satz zeichnete sich bereits ab, wie McClellands Leistungsmotivationstraining die neoliberale Forderung späterer Jahre nach mehr „individueller Eigenverantwortung“ unterfüttern half.^20^

## Strukturelle Ungleichheit als psychologisches Problem

Es läge nun nahe, aus McClellands Selbstdarstellung zu schließen, die gesellschaftliche Akzeptanz – und damit Wirkungsmacht – psychologischer Selbststeuerungstechnologien ergebe sich direkt aus deren Funktionieren. Auch der eingangs zitierte George A. Miller glaubte: „If our suggestions actually work, people should be eager to learn more.“^21^ Eben daraus hatte dieser ja die Forderung abgeleitet, Psychologen sollten ihr praktisch relevantes Wissen großzügig „weggeben“, um allen jenen zu helfen, die ihrem bedürften, was für ihn, wie wir sahen, jede und jeden meinte. Ein solches Narrativ unterschlägt allerdings den Aufwand, der mit einem solchen vermeintlich einfachen „Weggeben“ psychologischen Wissens auch innerhalb der USA verbunden war. Wichtiger noch: Es bringt zum Verschwinden, wie abhängig die Frage, ob ein derartiges Engagement auf Anklang stieß, von den orts- und zeitspezifischen Bedingungen war, unter denen es betrieben wurde. Ein Blick auf das von 1965 bis 1970 unter McClellands Leitung an mehreren Highschools in Massachusetts, Missouri und Kalifornien durchgeführte „Achievement Motivation Development Project“, soll dies abschließend veranschaulichen. Zu einer Zeit, als Leistungsunterschiede von Schüler*innen vielfach noch immer auf vermeintlich erblich bedingte IQ-Unterschiede zurückgeführt wurden – gestützt durch die Forschungen von Psychologen wie Arthur Jensen und Hans Jürgen Eisenck –, verband sich mit McClellands Ansatz das Versprechen, gerade im Bildungsbereich manifest werdende soziale Ungleichheiten überwinden zu können. Das Anfang der 1970er Jahre von ihm entwickelte Kompetenz-Konzept sollte als gerechtere Alternative den diskriminierenden und Lebenschancen verbauenden IQ-Test ersetzen und seit den 1980ern eine bemerkenswert weite Verbreitung finden – darauf sei an dieser Stelle nur verwiesen.^22^

Zu einem guten Teil ausgeführt durch *McBer & Company*, aber über die Harvard Universität beim U.S. Department of Health, Education, and Welfare als „Forschungsprojekt“ eingeworben, sollte das Projekt – ähnlich wie beim Unternehmertraining in Indien – erstens den Nachweis erbringen, dass sich auch die Leistungsmotivation von Schüler*innen würde steigern lassen, wenn man Lehrer*innen dazu ermunterte, ein leistungsförderliches Klassenklima herzustellen. Das hieß im Wesentlichen, so zu unterrichten, dass Schüler*innen die Gelegenheit bekämen, sich ihre eigenen Lernziele zu setzen, um so Selbständigkeit, Eigenverantwortung und Risikobereitschaft zu erlernen, mithin die Tugenden des Unternehmers, wie McClelland sie verstand. „[T]his training attempts to develop entrepreneurial ‚role responsibility‘. In theory this is consistent with the functions of schooling as an agent of socialization“, wie er in der Einleitung seines Abschlussberichts schrieb (McClelland & Alschuler 1971: 29). Zweitens und wichtiger ging es darum, Motivationstrainingsmaterialien zu erstellen und dann in Schulen zu verbreiten (McClelland & Alschuler 1971: 259). Nicht eigentlich die Generierung neuen Wissens war also das Ziel, denn dass Motivationstraining grundsätzlich funktioniere, war ja bereits vielfach gezeigt worden, sondern die Veränderung bestehender Curricula durch die Integration psychotechnischer Werkzeuge – aus Sicht des finanzierenden Departments of Health, Education, and Welfare letztlich eine Entwicklungsdienstleistung, kein Forschungsprojekt mit offenem Ausgang.

Was das Ziel der Verbreitung aber betraf, bekannte McClelland im Schlusskapitel seines Berichts, dass er Gespräche mit elf Schuldirektoren in Bostons *inner-city* hatte führen müssen, um nach einer „lengthy and sensitive period of explanation and negotiation“ (ebd.: 259 f.) schließlich drei von ihnen dazu zu bewegen, insgesamt etwa 60 Lehrer*innen an seinen Workshops teilnehmen zu lassen. Deren Programm bestand aus einer Selbstanalyse mit dem Ziel, die teilnehmenden Lehrer*innen ihre eigenen Werte reflektieren zu lassen. Anschließend bekamen sie die Funktionsweise und Erkennungsmerkmale des Leistungsmotivs erklärt. In einer dritten Phase wurden sie aufgefordert, zukünftige Unterrichtsstunden nach den erlernten Kriterien zu planen. McClelland nannte dies, Millers Formulierung aufgreifend, bezeichnenderweise ebenfalls „[to] giv[e] the findings of psychology away.“^23^

Aufgrund vielfältiger Verpflichtungen der meisten Lehrer*innen scheiterten die Programme letztlich aber an häufigen Unterbrechungen, Ablenkungen und fehlender Verstetigung der erprobten Strategien. Die ernüchternde Bilanz McClellands lautete: „it seems unlikely to us that the workshops had any major long-term effects“ (McClelland & Alschuler 1971: 261). Trainingskurse in Kalifornien blieben ähnlich erfolglos, nur ein Programm in Rensselaerville, New York, konnte zunächst Erfolge verbuchen, insofern die vorgeschlagenen Maßnahmen tatsächlich in den Unterricht integriert wurden. Die Einbeziehung der Schuldirektor*innen und damit der top-down Ansatz hatte es dort möglich gemacht.^24^ Aber auch hier fielen die Lehrer*innen schon bald in ihre alten Muster zurück. „[A]fter the research team left the school,“ räumte McClellend ein, „the ‚decay‘ curve set in“ (ebd.: 27). Mit anderen Worten: Psychologisches Training, das sich als Empowerment-Programm an Individuen wandte und gerade nicht auf die Veränderung von Strukturen abzielte, setzte funktionierende Strukturen voraus oder musste diese selbst schaffen, um erfolgreich zu sein. „It became apparent“, bilanzierte McClelland, „that a motivation program aimed at students must start with a diagnosis of the entire organizational context in which the teaching occurs“ (ebd.: 261).

Dieses Ergebnis entbehrte nicht einer gewissen Ironie, lief es doch der Argumentation zuwider, mit der McClelland und sein Kollege Alfred S. Alschuler die Notwendigkeit ihrer „Psychological Education“ begründeten, und zwar in den ersten Kapiteln desselben Berichts. Dort führte Letzterer aus, in den zurückliegenden Jahren sei zunehmend klar geworden, dass gerade strukturelle Veränderungen nicht ausreichen würden, um Probleme wie Rassismus, Aggression und Schulversagen zu beheben. Alschuler verwies zur Untermauerung dieser Behauptung auf einen Bericht, der wie kein zweiter seit seiner Veröffentlichung im Jahr 1966 in der amerikanischen Öffentlichkeit für Diskussion sorgte, nämlich den „Equality of Educational Opportunity“ Bericht von James S. Coleman, kurz Coleman-Report genannt. „The Coleman Report […] has shown“, schrieb Alschuler, „that student attitudes towards themselves and about the responsiveness of the world to their efforts are more strongly related to academic gains than differences in curricula, facilities or teacher training“ (ebd.: 37). Tatsächlich war Coleman zu dem für viele überraschenden Schluss gekommen, dass nicht die Ausstattung einer Schule, die Qualität der Lehrer, die Zusammensetzung der Schülerschaft, sondern der familiäre Hintergrund der entscheidende Faktor für schulische Leistung sei. Dieser Hintergrund determiniere das Mindset der Schüler*innen und damit ihre Grundeinstellung zu Lernen und Bildung (Coleman 1966: 297). Alschulers Verweis auf dieses Ergebnis machte es möglich, „Psychological Education“ gleichsam als letzte noch verfügbare Alternative hinzustellen.^25^

Alschuler und McClelland verwiesen aber noch auf ganz andere Faktoren, um die Notwendigkeit ihres Ansatzes plausibel zu machen. So hätte die zunehmende Zahl von Studenten- und Schülerprotesten der letzten Jahre sowie die „Rassenunruhen“ und öffentlichen Gewaltausbrüche gezeigt, dass gleichsam die ganze Nation eine psychologische Behandlung benötige. Nicht nur könne dieser Bedarf schon längst nicht mehr von den vorhandenen Psychotherapeuten abgedeckt werden, die klassische Psychotherapie sei mit ihrer Vergangenheitsfixiertheit auch völlig ungeeignet, hier zu helfen.^26^„[N]ew methods are needed to promote psychological growth, thus preventing these human problems from occuring and making remediation unnecessary“ (ebd.). Proteste, Gewalt, Aufruhr und die Mordanschläge der letzten Jahre seien für die „Psychological Education“ das, was der Sputnikschock in den zurückliegenden zehn Jahren für die Einführung neuer akademischer Curricula gewesen war: eine die Nachfrage ungemein steigernde Anregung.^27^ Psychologisch geschulte Erzieher und Pädagogen, die bislang isoliert voneinander gearbeitet hätten, kämen vermehrt zusammen und begönnen zu kooperieren. Neue Zentren entstünden, die ihre auf psychologische Entwicklung ausgerichteten Kurse der breiten Öffentlichkeit zugänglich machten (ebd.). Bei den (nur zum Teil) neuen Zentren für Psychologische Bildung, an die Alschuler hier dachte und auf die er in mehreren Fußnoten auch verwies, handelte es sich um die National Training Laboratories in Bethel, Maine, das Western Behavioral Sciences Institute in La Jolla, Kalifornien, Outward Bound, Inc. in Andover, Massachusetts, und vor allem das Esalen Institut an der Küste von Big Sur, Kalifornien. Das *Life Magazine* hatte im Juli 1968 die dort stattfindenden Kurse in einer 15seitigen Reportage ausführlich beschrieben (Alschuler 1969: 1 f.). Hier übten sich experimentierfreudige Menschen unter der Leitung von Psychologen wie Carl Rogers und Abraham Maslow in der Kommunikation ihrer wahren Gefühle, um eine neue, authentischere Lebensform zu erproben (Weidman 2016: 122–128). Indem Alschuler in seinem Bericht auf den *Life*-Artikel verwies, stellte er einen direkten Zusammenhang zwischen den gravierenden gesellschaftlichen Missständen im Land und dem seit Mitte der 1960er Jahre immer mehr Zulauf erhaltenden „Human Potential Movement“ her. Dessen prominentester Sprecher, Michael Murphy, erklärte: „We don’t make sick people well but well people better“ (Howard 1968: 56).

Das „Achievement Motivation Development Project“ aufgrund dieser Verweise als Teil der gegenkulturellen Bewegung zu sehen, wäre allerdings verfehlt. Nicht nur stand McClelland den Arbeiten Abraham Maslows skeptisch gegenüber – er hielt sie schlicht für unwissenschaftlich, während sich dieser seinerseits von der akademischen Psychologie distanzierte.^28^ Die psychologischen Gurus der Gegenkultur wie Timothy Leary oder Richard Alpert, alias Ram Das – ein ehemaliger Schüler McClellands an der Wesleyan Universität –, rechneten McClelland und dessen Forschung selbst zu ihren Antagonisten.^29^ McClellands und Alschulers Verweise auf das Human Potential Movement half jedoch, Dringlichkeit und Nachfrage ihrer Programme gegenüber den geldgebenden Institutionen plausibel zu machen.

Zwar war es McClelland und seinem Team Anfang der 1970er Jahre gelungen, ihr Wissen auch in Form von Manualen, Arbeitsbüchern, Lehrfilmen und einigen Workshops weiterzuverbreiten. Über 3.000 Exemplare des eigenen ‚Teaching Achievement Motivation‘-Manuals, 7.000 Arbeitsbücher für Schüler*innen sowie diverse selbst produzierte Lehrfilme wurden über den Verlag „Educational Ventures“ verkauft (McClelland & Alschuler 1971: 264). Dies hieß aber nicht, dass die „Psychological Education“ Eingang in die Curricula von Schulen gefunden hätte und in diesem Sinn gesellschaftlich erfolgreich gewesen wäre. In einem unveröffentlichten Interview mit Isabel Grossner vom Oral History Research Office der Columbia University bekannte McClelland 1983 ernüchtert: „[I]f I were to be honest to the guy that gave the money, I don’t think the payout for education has been worth it so far. I hate to say that, but I really don’t think so.“^30^ Für sein Beratungsunternehmen waren Schulen als Klienten damit nicht mehr länger lukrativ.^31^

Hatte McClelland 1963 die Human Resources Development Corporation (ab 1965 McBer & Company) ins Leben gerufen, um sich von den administrativen Zwängen der Universität und vor allem den Unwägbarkeiten forschungsfördernder Geldgeber zu befreien, so erwies sich ihm der Beratungsmarkt zwar auch als unsicher, insofern er dort von der jeweils aktuellen Nachfragesituation abhängig wurde, aber doch als kalkulierbar. Ab den 1970er Jahren richtete McClelland seinen Forschungsfokus darum auf ein Thema aus, mit dem zahlungskräftigere Klienten adressierbar wurden: das Machtmotiv (vgl. McClelland 1970: 31). Wie Matthew Hoffarth gezeigt hat, wurde das Machtmotiv in den 1970er Jahren McClellands Markenzeichen, mit dem McBer & Company Manager von Großunternehmen wie Aviation Corporation of America, Boston Gas Company, Hotel Corporation of America oder die New England Merchants National Bank erreichte (Hoffarth 2020: 165). In einer Zeit zurückgehender staatlicher Fördermittel für sozialwissenschaftliche Forschung insgesamt hatte sich McClelland damit eine Einnahmequelle erschlossen, die fortan reichlich sprudeln sollte.^32^ McBer & Company erwartete 1986 einen geschätzten Umsatz von 5 bis 10 Millionen Dollar (Kennedy & Kennedy Burke 1987: 29).

## Fazit: Politik der Selbststeuerung in den langen 1960er Jahren

Der Versuch, Gesellschaft vom Individuum ausgehend zu gestalten, setzte nicht das Scheitern von strukturverändernder Planungspolitik voraus, er wurde in den langen 1960er Jahren gleichzeitig mit dieser unternommen. David McClelland präsentierte seinen auf individuelle Selbststeuerung setzenden Zugang erstmals 1957, im selben Jahr, in dem Walt Rostow und Max Millikan mit ihrer Schrift *A Proposal: Key to an Effective Foreign Policy* das modernisierungstheoretische Denken der zurückliegenden Jahre für die Eisenhower-Administration aufbereiteten.^33^ Und so wie die Effekte dieser modernisierungstheoretischen Planungspolitik erst mit dem Antritt der Kennedy-Administration zum Tragen kamen, begann McClelland auch erst im Sommer 1961 seine Ideen eigenhändig ins Werk zu setzen – erst in Mexiko, dann in Indien, schließlich in vielen weiteren Ländern.

Amy C. Offner hat darauf hingewiesen, dass David Lilienthal, der ehemalige Leiter der Tennessee Valley Authority, den Ausdruck „social entrepreneurship“ bereits 1966 prägte, ein Konzept, das fälschlicherweise, wie sie schreibt, mit dem späten 20. Jahrhundert in Verbindung gebracht wird, sich aber nicht als Reaktion auf einen desillusionierten Planungsoptimismus herausbildete, sondern Ausdruck einer dazu parallel betriebenen Entwicklungspraxis war (Offner 2019: 182 f.). Dazu passt, dass McClellands Politik der Selbststeuerung, die er ab Mitte der 1960er Jahre auch innerhalb der USA zur Anwendung brachte, auf sein aktives unternehmerisches Engagement angewiesen war. Denn weder folgte aus dem Funktionieren seiner psychologischen Technologien schon deren gesellschaftsweite Akzeptanz, noch wurden Großprobleme wie Armut und soziale Ungleichheit von sich aus als individuell lösbar gedeutet. Psychologen, die wie McClelland in die Gesellschaft hineinzuwirken wünschten, mussten ihr Wissen erst in Trainingsprogramme übersetzen und anschließend selbst für deren Verbreitung sorgen. Das aktivistische Anliegen, Gesellschaft zu gestalten, wurde so zugleich zu einer unternehmerischen Tätigkeit.

Als probates Mittel gesellschaftlicher Intervention überzeugte psychologisches Wissen aber nur so lange, wie sich die kollektive Arbeit am Individuum – die Ermächtigung zur Selbststeuerung – gegenüber der Arbeit an den Strukturen profilieren ließ. Ob das gelang, war weniger eine Frage der Rhetorik als der Umstände, auf die dabei verwiesen werden konnte. In außenpolitischer Hinsicht kam McClelland hier der Wettlauf um Einflusssphären im Globalen Süden entgegen, weil dieser Wettlauf für alternative Entwicklungsideen empfänglich machte. Innenpolitisch spielten die Unruhen der 1960er Jahre den Verfechtern psychotechnischer Ansätze in die Hände, insofern man angesichts des anscheinenden Unvermögen des Staates, gesellschaftliche Ordnung zu garantieren, auch hier nach Maßnahmen zu suchen begann, deren disziplinierende Wirkung weiter reichen würde als staatliche Verordnungen es je könnten – nämlich bis in die Psyche des Einzelnen hinein. Das gesellschaftliche Interesse an „Psychological Education“ – festzumachen an der Gründung entsprechender Zentren und der Verbreitung entsprechender Literatur – war eine Reaktion auf diese „Krise des Regierens“ und damit prekärer – weil von diesem Umstand abhängig –, als jene meinen, die der Angewandten Psychologie einen weitreichenden Einfluss zusprechen, „strukturelle Probleme hochentwickelter Industriegesellschaften so zu interpretieren, dass ihre Lösung nicht durch politisches Handeln, sondern durch psychologische Behandlung zu erwarten ist“ (Gelhard 2018: 9; vgl. auch Rose 1998: 83). Eine solche Beschreibung bleibt den Selbstdarstellungen der beteiligten Psychologen verhaftet, die den Erfolg ihrer Programme eher an deren Charakter als geglückte Experimente messen als an einer sich tatsächlich verändernden Gesellschaft. Das zeigte sich an McClellands praktischen Erfahrungen sowohl in Indien als auch in den USA, wo seine Trainingsprogramme für Unternehmer*innen und Lehrer*innen an ihrer ausbleibenden Verstetigung politisch scheiterten, während sie zugleich, wie er auch später immer wieder betonte, als psychologische Pilotstudien erfolgreich waren. „After the project ended“, schrieb McClelland beispielsweise über seinen Einsatz in verschiedenen amerikanischen Schulen rückblickend, „the teachers were moved on to a major new reading program that was federally funded, despite impressive evidence of the success of [the] training“ (McClelland 1978: 207).

McClelland tauschte die Launen der Förderpolitik nicht zuletzt deshalb gegen die Risiken des Marktes ein und wurde zum Unternehmer-Wissenschaftler, weil ihm für Nachfrage auf dem sich weitenden Beratungsmarkt zu werben einfacher schien als politische Entscheidungsträger*innen von der Wirksamkeit seiner Programme zu überzeugen. Politikern leuchtete zwar grundsätzlich ein, dass Leistungsmotivation eine wünschenswerte Eigenschaft sei, nicht aber McClellands daraus abgeleiteter Anspruch, ganze Gesellschaften entwickeln zu können – nicht, mit anderen Worten, sein entwicklungspolitischer Ansatz. Zu spüren bekam McClelland dies, als er 1967 vor einem Unterausschuss des Committee on Foreign Affairs im amerikanischen Repräsentantenhaus als geladener Experte sprach. „I agree with what you say about motivation“, entgegnete ihm dort ein Abgeordneter trocken, „except when you come to supply it and then I fall down completly“ (McClelland 1967: 83–122, hier 119). Selbst der Journalist T. George Harris, der McClelland 1971 für *Psychology Today* interviewte, bezweifelte, dass Regierungen dessen motivationspsychologischem Entwicklungsansatz jemals folgen würden. Politiker würden sich letztlich vor Psychologen fürchten und öffentliche Institutionen seien generell zu ungeschickt, um mit Motiven, Phantasien und all jenem umzugehen, was sich im Inneren der Menschen abspiele. McClelland antwortete darauf: „[W]e’ve been trying to do it outside of government. Maybe entrepreneurs can develop the entrepreneurial motive around the world“ (Harris 1971: 74).

Der Anti-Institutionalismus, der aus dieser Haltung sprach, war in den späten 1960er, frühen 1970er Jahren freilich weder neu noch einzigartig. Soziale Bewegungen zur Abschaffung von Gefängnissen, psychiatrischen Einrichtungen und zur Entschulung der Gesellschaft schlugen viele in ihren Bann (Richert 2019; Scull 1980). McClelland verstand aus dieser kritischen Grundstimmung Kapital zu schlagen. Was ihm als beratender Experte verwehrt blieb, erreichte er als Unternehmer-Wissenschaftler. Das war nicht zuletzt deshalb so, weil die Skepsis von Politikern gegenüber psychotechnischen Strategien Regierungsbehörden nicht davon abhielt, bei der Umsetzung ihrer politischen Leitlinien – in den 1960er Jahren des *War on Poverty* – auf jene Trainingsangebote zurückzugreifen, die Unternehmen wie *McBer & Company* als Mittel zur Armutsbekämpfung innerhalb der USA priesen (vgl. dazu auch Moran 2018). Aus Beratersicht war der Staat so ein zahlungskräftiger Konsument psychotechnischer Angebote wie andere Großunternehmen auch. So vergab Ende der 1960er Jahre etwa die Economic Development Administration Trainingsaufträge an das von McClelland mitgegründete Behavioral Science Center zur Schulung von schwarzen Kleinunternehmern (McClelland 1969c: 6), Boston’s „poverty agency“ hatte einen Vertrag mit Training Development Systems, ein ähnliches Unternehmen, das sich auf Programme für Drogenabhängige spezialisierte (siehe Harris 1971: 75). Das Office of Economic Opportunity bestellte sich bei McClelland „power-motivation training“ (ebd.). Was die Wirksamkeit dieser Programme anlangt, so kam eine Studie 1976 zu ähnlich gemischten Ergebnissen wie schon McClelland in Indien: Manche der Geschulten wurden aktiver, viele aber auch frustrierter. „Part of this frustration may have stemmed from deteriorating economic conditions beyond their control“, hieß es dort zur Erklärung lapidar (Durand 1975: 88). Wie ich zu zeigen versucht habe, waren Programme wie diese schon in den 1960er Jahren – ob sie nun funktionierten oder nicht – Teil dessen, was wir als staatlich gesteuerte Wohlfahrtspolitik erinnern. Als in den 1970er und 1980er Jahren die kapitalistischen Ökonomien in die Krise gerieten, konnte bei der Neuordnung des politisch-ökonomischen Systems auf Kontakte und Werkzeuge zurückgegriffen werden, die man davor bereits erprobt hatte (Offner 2019: 278 f.). Das war weniger ein „Durchbruch“ neuer (neoliberaler) Ideen als eine Vereinseitigung einer bereits betriebenen Praxis. Die sogenannte neoliberale Ära, so ließe sich davon verallgemeinernd ableiten, wurzelt nicht nur im Denkkollektiv der Mont Pèlerin Society, sie geht auch auf Akteure zurück, die sich selbst gar nicht als Neoliberale verstanden.

## Footnotes

1 Explizit zu „Antipolitik“ aus politologischer Perspektive (Schedler 1997).

2 Im westdeutschen Kontext ist häufig von „Globalsteuerung“ die Rede, ein Begriff, der vor allem mit dem Wirtschaftsminister Karl Schiller und dessen „Stabilitätsgesetz“ von 1967 in Zusammenhang steht, zuweilen aber auch – etwas schief – auf die „New Economics“-Politik der Kennedy-Administration angewendet wird (etwa bei Schatz 1988: 188). Zu den Unterschieden zwischen westdeutscher „Globalsteuerung“ und amerikanischem Keynesianismus, siehe die vergleichenden Analysen von Dickhaus (2001) und Hagemann (2001).

3 Siehe dazu Latham (2011: 159–162); Flamm (2005: 1–11); Lepore (2018: 610–645).

4 David McClelland: *Additional notes on the proposal for research on ‚Achievement Motivation and economic development‘* (August 7, 1955), S. 3, Grant 05500211, Reel 17, Ford Foundation Archives, Rockefeller Archive Center, Sleepy Hollow, New York.

5 Confidential Docket Excerpt. Board of Trustees Meeting (September 30, 1955), Behavioral Science Research Grants, Grant 05500211, Reel 17, Ford Foundation Archives, RAC.

6 So etwa Hans Jürgen Eysenck, siehe Eysenck (1952). *Zur dominanten Einschätzung in der Psychologie, Erwachsene könnten nicht dazu gebracht werden, neue Motive zu erwerben* (McClelland & Winter 1969: 39 ff.).

7 Es handelte sich um Russell William Burris, der unter der Anleitung B. F. Skinners an der Indiana University promovierte, siehe Burris (1958) (unveröffentlichte Dissertationsschrift), siehe den kurzen Bericht zu Burris Vorgehen bei Kolb (1965: 784).

8 Siehe dazu Ferster (2014: 50–92).

9 Siehe auch den früheren Verweis auf den Einfluss des Behaviorismus in McClelland (1965a: 322).

10 Siehe zur Chronologie der Pilotstudien den kurzen historischen Abriss in: *Motivation Development for Management.* Programme by The Centre for Research in Personality, Harvard University, USA, Background, S. 3, McClelland papers, Archives of the History of American Psychology, Cummings Center for the History of Psychology—The University of Akron (im Folgenden abgekürzt als CCHP), box M2890, folder 7.

11 *Motivation Development for Management.* Programme by The Centre for Research in Personality, Harvard University, USA, Background, S. 5.

12 David C. McClelland: *Research proposal for a study of the effects of motivationally-oriented management development courses on rate of economic growth in underdeveloped countries* (07.01.1963), S. 3, David C. McClelland papers, Archives of the History of American Psychology, CCHP, box M2888, folder 4.

13 Siehe McClellands Bericht (McClelland & Winter 1969: 96). McClelland nutzte die Elektrizitätsproduktion als Maß für wirtschaftliche Produktivität (statt des üblicheren Bruttoinlandsprodukts), weil er der Meinung war, Kilowattstunden ließen sich international leichter vergleichen.

14 David C. McClelland: *Research proposal for a study of the effects of motivationally-oriented management development courses on rate of economic growth in underdeveloped countries* (07.01.1963), S. 3.

15 „[We] could run the whole project through Harvard,“ schrieb er im Januar 1963 in einem Brief, „but I think it would be very inconvenient and would involve me in more administration than I want to get into.“ Brief David C. McClellands an Dr. Birtill A. Lloyd (11.01.1963), S. 2, David C. McClelland papers, Archives of the History of American Psychology, CCHP, box M2888, folder 4.

16 Brief von David E. Bell, Office of the Administrator, Department of State, Agency for International Development an David C. McClelland (09.10.1963), David C. McClelland papers, Archives of the History of American Psychology, CCHP, box M2894, folder 3.

17 Draft of letter to David Bell (nicht datiert, wahrscheinlich November 1963, handschriftlicher Vermerk „not sent“), S. 5, David C. McClelland papers, Archives of the History of American Psychology, CCHP, box M2888, folder 4 (gezeichnet von David C. McClelland), (ebd.: 107).

18 „The problems were much the same with the Ford Foundation (…).“ (McClelland & Winter 1969: ix).

19 Siehe die Hinweise bei McClelland & Dowling (1972: 63 f.); Berlew & LeClere (1974): Miron & McClelland (1979); siehe auch „Trips of McClelland“ und „Curriculum Vitae“, Papers of David McClelland, Harvard University Archives, HUGFP 145, Box 25, Folder: Curriculum Vitae [and other biographical information, 1955–1974].

20 Zum Prinzip der individuellen Selbstverantwortung beispielsweise bei Milton Friedman, siehe Burgin (2012: 189ff.).

21 „If our suggestions actually work, people should be eager to learn more.“ (Miller 1969: 1072 und 1073).

22 Siehe dazu Gelhard (2018).

23 McClelland gebrauchte diese Formulierung im Vorwort zum ein Jahr zuvor erschienenen „Teaching Achievement Motivation“-Manual, das die Ergebnisse des ‚Achievement Motivation Development Projects‘ zu einem do-it-yourself-Trainingsbuch für Lehrer aufbereitete. Wörtlich heißt es dort: „Another unusual characteristic of this book is that *it gives the findings of psychology away*, so to speak.“ (Alschuler et al. 1970: xiv).

24 „Apparently introducing training at the top of the school systems (for administrators) has more long-range impact than introducing it at the bottom (for pupils).“ (Ebd.: 262).

25 Siehe zur Geschichte und Wirkung des Coleman-Reports (Alexander & Morgan 2016: 1 ff.).

26 „From this perspective psychotherapy is not so much wrong but inappropriate to alleviate widespread racism, aggression, interpersonal insensitivity, moral irresponsibility, and non-self-actualization.“ (McClelland & Alschuler 1971: 36).

27 „Protests, violence, riots and assassinations are doing for Psychological Education what Sputnik did to spur the new academic curricula in the last ten years.“ (Ebd.: 32).

28 Vgl. McClelland (1955: 159).

29 „The psilocybin group certainly perceived me as one of their chief antagonists“, hielt McClelland in einem Brief an Elliott Danzig fest. Brief David C. McClellands and Elliott Danzig, 6. Oktober 1964, David C. McClelland papers, Archives of the History of American Psychology, CCHP, box M2888, folder 4.

30 Oral History Research Office, Columbia University: The Reminiscences of David C. McClelland, S. 21, (New York 1983). Harvard University Archive, McClelland Papers, Box 25 [Spencer Foundation Oral History Project. The Reminiscences of David C. McClelland—1983].

31 „You see“, gab er im selben Interview zu Protokoll, „a consulting firm gives it up because there isn’t any money in the schools. They don’t have enough to pay a consulting firm.“ The Reminiscences of David C. McClelland, S. 21.

32 Zum Rückgang staatlich geförderter Forschung in den 1970er Jahren, siehe Leslie (1993: 1).

33 Vgl. Lorenzini (2019: 61).
